# The Impact of AI-Enabled Job Characteristics on Manufacturing Workers’ Work-Related Flow: A Dual-Path Perspective of Challenge–Hindrance Stress and Techno-Efficacy

**DOI:** 10.3390/bs15101320

**Published:** 2025-09-26

**Authors:** Hui Zhong, Yongyue Zhu, Xinwen Liang

**Affiliations:** School of Management, Jiangsu University, Zhenjiang 212013, China; 2212310041@stmail.ujs.edu.cn (H.Z.); zhuyy@ujs.edu.cn (Y.Z.)

**Keywords:** AI-enabled job characteristics, perceived challenge stress, perceived hindrance stress, work-related flow, techno-efficacy

## Abstract

The integration of artificial intelligence (AI) in the manufacturing industry is increasingly prevalent, presenting both ongoing opportunities and challenges for organizations while also significantly impacting worker behavior and psychology. Drawing on data from 405 workers in China, this study employs hierarchical regression analysis and fuzzy-set qualitative comparative analysis (fsQCA) to investigate the influence mechanism of AI-enabled job characteristics on work-related flow. Key findings reveal that: AI-enabled job characteristics positively predict work-related flow by increasing perceived challenge stress, yet simultaneously exert a negative influence by exacerbating perceived hindrance stress; techno-efficacy significantly alleviates the relationship between AI-enabled job characteristics and perceived hindrance stress but does not moderate the path via perceived challenge stress; fsQCA identifies four distinct causal configurations of antecedents leading to high work-related flow. This research elucidates the complexities of AI-enabled job characteristics and their dual-faceted impact on work-related flow. By integrating AI into the study of worker psychology and behavior, it extends the contextual scope of job characteristics research. Furthermore, the application of fsQCA provides novel insights into the antecedent conditions and configurational pathways for achieving work-related flow, offering significant theoretical and practical implications.

## 1. Introduction

The pursuit of meaning and positive experience at work is a growing trend in the 21st-century workforce, with work-related flow—a state of deep immersion, valued experience, and intrinsic joy (Bakker, 2005)—being a key contributor to worker motivation and happiness (Datu & Mateo, 2017). This trend is particularly evident among the rising generations of workers (e.g., Millennials and Generation Z), who often prioritize engaging tasks and personal fulfillment (Oerlemans & Bakker, 2018). Their heightened emphasis on self-awareness and meaningful achievement (Li, 2017) underscores that fostering work-related flow has become increasingly critical for worker satisfaction and retention in the modern era (Salanova et al., 2011).

However, the manufacturing sector has traditionally presented a challenging environment for fostering such positive experiences. Characterized by repetitive tasks, heavy workloads, and often suboptimal conditions (Khaw et al., 2019), conventional manufacturing roles have been more associated with burnout and reduced well-being than with flow (Y. Zhu et al., 2020). The continuous and pervasive reforms driven by artificial intelligence in all industries serve to radically reshape the prevailing context (Cui et al., 2024). Technologies such as machine learning and smart logistics are not only altering production processes but also fundamentally redefining the very nature of manufacturing jobs (Mirbabaie et al., 2022; Zhai & Liu, 2023).

While AI introduces possibilities for more intelligent and adaptive operations (Jayan et al., 2025), it also imposes new cognitive and emotional demands, often exceeding workers’ established resources and even threatening job security (Cavicchioli et al., 2025). Understanding how manufacturing workers adapt to these AI-enabled job characteristics is therefore critical. This adaptation affects not only the experience of work-related flow but also the well-being of the workforce and the ultimate success of industrial upgrading initiatives.

Since job characteristics drive workplace stress (Zhao et al., 2016)—triggering varied perceptions and coping responses (Abbas & Raja, 2019)—this study examines how AI-altered job traits affect manufacturing workers’ flow through a stress lens. Drawing on work design theory (Humphrey et al., 2007; Garcia Martinez, 2017), we focus on task characteristics (autonomy, skill variety) and knowledge characteristics (complexity, specialization, information processing) (Verma & Singh, 2022). These dimensions reflect digitalization’s impact on work design (Schroeder et al., 2021). Guided by cognitive appraisal theory, we propose AI job traits evoke: Challenge stress (fostering growth and positive affect) or Hindrance stress (triggering anxiety) (Folkman et al., 1986). Critically, individual differences shape these appraisals. Techno-efficacy—confidence in one’s ability to use technology—serves as a key filter in AI contexts (Liu et al., 2024). High techno-efficacy helps workers reframe AI demands as manageable challenges, buffering hindrance stress. Low techno-efficacy amplifies threat perceptions. We thus integrate it as a moderator to clarify boundary conditions.

Although AI-driven transformation is pervasive in manufacturing, scholarly understanding of its impact on workers in this sector is still developing. This research addresses:

How do AI-enabled job characteristics influence work-related flow?

Does the challenge–hindrance stress framework mediate this relationship?

How does techno-efficacy moderate stress appraisal paths?

Therefore, this study aims to bridge this gap by investigating the dual-path mechanism of AI-enabled job characteristics on work-flow, and the moderating role of techno-efficacy. Our analysis of data from 405 manufacturing workers in China reveals several key findings. First, we confirm that AI-enabled job characteristics exert a dual effect on work-flow: they enhance it by increasing perceived challenge stress, but also inhibit it by increasing perceived hindrance stress. Second, techno-efficacy serves as a critical boundary condition, mitigating the relationship between AI-enabled job characteristics and hindrance stress. Finally, our fsQCA results further identify that a combination of long job experience, high AI-enabled job characteristics, and high techno-efficacy is sufficient for achieving high work-flow. These findings will advance context-specific understanding of job characteristics’ psychological impacts and guide manufacturing management during AI integration, which will be discussed in the subsequent sections.

## 2. Theoretical Basis and Research Hypothesis

### 2.1. Cognitive Appraisal Theory of Stress

Cognitive appraisal theory of stress elucidates individual differences in stress responses by emphasizing that stress stems not directly from stressors but from individuals’ subjective appraisals (Lazarus & Folkman, 1987). Distinct cognitive evaluations generate divergent stress perceptions, subsequently shaping coping behaviors. This appraisal process involves two sequential stages: Primary appraisal classifies stressors as either perceived challenge stress (interpreted as growth opportunities) or perceived hindrance stress (viewed as threatening constraints). Secondary appraisal then assesses response options, including the feasibility of environmental change, necessity of acceptance, and one’s self-regulation capacity. Within AI-driven workplaces, this theory clarifies why workers experience identical job characteristics differently. AI-induced stressors interact with worker cognition, generating challenge–hindrance perceptions that either enhance or undermine work-related flow (X. Zhu et al., 2021).

### 2.2. AI-Enabled Job Characteristics and Perceived Stress

Job characteristics describe the nature of work and its related attributes. This concept traces back to Taylor’s “scientific management” principles: work specialization, systematization, simplification, and standardization. Hackman and Oldham (1975) later developed the Job Characteristics Model (JCM), identifying five dimensions: autonomy, task identity, task significance, skill variety, and feedback. These dimensions shape workers’ psychological states. Recent studies extend the JCM to address new societal trends. For example, Lu et al. added “digital literacy” to analyze farmers’ pro-environmental behaviors (S. Lu et al., 2024b), while Astakhova et al. (2024) found job characteristics’ motivational effects vary by occupation and passion type (harmonious vs. obsessive).

As manufacturing firms adopt AI, traditional JCM struggles to explain technology’s psychological impact. The model was conceived in an era of predominantly human-operated work, and its dimensions do not fully capture the nuances of human-AI collaboration. For instance, ‘autonomy’ becomes complicated when shared with or constrained by AI systems’ decision-making protocols (Parker & Grote, 2022). Similarly, ‘feedback’ may become continuous, immediate, and algorithmically generated (Kellogg et al., 2020), potentially leading to surveillance stress rather than developmental growth. The very nature of ‘skill variety’ is transformed as AI automates routine tasks and introduces new demands for AI management and interpretation skills (Autor, 2015).

In response to technology’s impact, Carlson et al. (2017) proposed a technology-driven JCM, which shifts the focus to the technological environment itself, examining how characteristics like technological autonomy, overload, and monitoring affect general work attitudes. In contrast, Verma and Singh (2022) framework of AI-enabled job characteristics offers a more direct evolution of the original JCM. It reconceptualizes the core job characteristics specifically for the AI context, categorizing them into task-related dimensions (Work Autonomy, Skill Variety) and knowledge-related dimensions (Work Complexity, Specialization, Information Processing).

The key difference lies in their theoretical focus: Carlson et al.’s model addresses the broader technological context as a source of stressors or facilitators, while Verma and Singh’s model delves into how the fundamental nature of the job tasks and knowledge requirements are transformed by AI. For this study, which aims to understand how the intrinsic characteristics of AI-enabled jobs influence worker psychological states, Verma and Singh’s (2022) approach was chosen. Their framework provides a more granular and directly applicable lens for analyzing the specific dimensions of jobs that are redesigned through AI integration, making it particularly suitable for investigating the psychological mechanisms in modern manufacturing settings (Oldham & Fried, 2016).

These findings confirm that technology reshapes job characteristics, making context-specific refinements essential (Venkatesh et al., 2010). Accordingly, this study adopts Verma and Singh’s framework for job characteristics, categorizing them into five dimensions: AI-enabled Job Autonomy, AI enabled Skill Variety, AI enabled Job Complexity, AI enabled Specialization, AI enabled Information Processing.

Research shows job characteristics can cause both positive and negative stress, depending on workers’ cognitive appraisal (Daniels, 2006). It is noteworthy that the same AI-enabled job characteristics may simultaneously induce both challenge and hindrance stress, and individuals may even experience high levels of both types of stress reactions (Rodell & Judge, 2009). Their specific manifestations depend on the combined effects of individual differences and contextual factors (Lazarus & Folkman, 1987). When workers view AI-driven changes as a challenge, greater autonomy and skill variety boost role flexibility, enhancing perceived competence. Specialization and information processing also streamline decisions, reducing uncertainty and improving efficiency. This challenge stress fosters positive outcomes like work happiness (Xu et al., 2023), green behavior (Waqas et al., 2021), and creativity (Tu et al., 2023).

Conversely, if workers perceive AI changes as a threat, they experience hindrance stress. As Garcia Martinez (2017) notes, AI work demands skills like critical thinking, creativity, and data analysis, rapidly evolving skill requirements and complexity may trigger negative self-evaluations and job insecurity. Thus, the influence of AI-enabled job characteristics on stress perception possesses a “double-edged sword” attribute (J. A. LePine et al., 2004): it can be perceived either as a challenge or as a hindrance, depending on individual cognition and available resources. Based on this, we hypothesize the following:

**H1a:**  *AI-enabled job characteristics are positively related to perceived challenge stress.*

**H1b:**  *AI-enabled job characteristics are positively related to perceived hindrance stress.*

### 2.3. Perceived Challenge Stress, Perceived Hindrance Stress, and Work-Related Flow

Work-related flow describes short-term peak experiences during work (Bakker, 2008). It comprises three dimensions: (1) Concentration: A state of complete focus where workers immerse themselves in work, blocking out external distractions. (2) Work enjoyment: The pleasure and happiness derived from work, resulting from cognitive and emotional engagement in the flow state. (3) Intrinsic work motivation: Motivation originating from the work itself rather than external rewards. Workers driven by intrinsic motivation become deeply absorbed in tasks and seek to sustain their engagement.

The research of Tomaka et al. (1997) indicates that perceived challenge stress and hindrance stress may coexist in certain situations due to counteracting physiological responses. However, only one type typically dominates. When challenge stress predominates, workers’ engagement and sense of achievement intensify (Karatepe et al., 2021). This motivates greater energy investment in work, facilitating work-related flow. Conversely, under hindrance stress, workers perceive their work as threatening to growth and development (Sawhney & Michel, 2022). This elevates psychological strain, triggering anxiety, burnout, and negativity. Consequently, affected workers struggle to concentrate on tasks or derive joy from work.

Therefore, we hypothesize the following:

**H2a:**  *Perceived challenge stress is positively related to work-related flow.*

**H2b:**  *Perceived hindrance stress is negatively related to work-related flow.*

### 2.4. The Mediating Role of Perceived Challenge and Hindrance Stress

According to stress cognitive appraisal theory, individuals perceiving stress as a challenge typically adopt positive coping strategies, whereas those experiencing hindrance stress tend toward negative coping strategies (Folkman et al., 1986). Specifically, when workers interpret job characteristics as a challenge, this challenge stress fosters future growth, rewards, and personal benefits (Kundi et al., 2022). It stimulates intrinsic motivation and achievement, particularly when workers believe they can manage the stress. This leads to positive outcomes including enhanced performance (Mazzola & Disselhorst, 2019), job satisfaction (Y. Zhang et al., 2019), and creativity (L. Zhang et al., 2016). Consequently, challenge stress maintains high work engagement, enabling deep immersion in tasks.

Conversely, viewing job characteristics as a hindrance reflects negative person-environment interactions. Workers may perceive their abilities as inadequate to cope with stressors or view stress as detrimental to well-being (Casper & Sonnentag, 2020). This results in exhaustion and diminished task engagement, manifesting as avoidance behaviors, reluctance to pursue challenges, and reduced potential utilization. Howard et al. (2020) demonstrate that handling such obstacles amplifies individual stress, causing learning resistance and counterproductive work behaviors. Empirical evidence further confirms hindrance stress triggers anger and emotional exhaustion, ultimately contributing to burnout (Aman-Ullah et al., 2025), job insecurity (Cao & Song, 2025), reduced engagement (Curran & Prottas, 2017), and impaired work-related flow attainment.

Integrating H1a,b and H2a,b, this study posits that job characteristics affect work-related flow through perceived challenge and hindrance stress. The following mediation hypotheses are proposed:

**H3a:**  *Perceived challenge stress mediates the relationship between AI-enabled job characteristics and work-related flow. AI-enabled job characteristics are positively related to work-related flow via perceived challenge stress.*

**H3b:**  *Perceived hindrance stress mediates the relationship between AI-enabled job characteristics and work-related flow. AI-enabled job characteristics are negatively related to work-related flow* via *perceived hindrance stress*.

### 2.5. The Moderating Role of Techno-Efficacy

Techno-efficacy—defined as an individual’s self-assessment of their ability to use technology for task completion, including evaluations of available resources for adopting new technologies (Liu et al., 2024)—has gained scholarly attention with workplace AI implementation. Workers with high techno-efficacy typically select challenging tasks, set ambitious goals, stimulate intrinsic motivation, and immerse themselves in work (Knight et al., 2017). Conversely, those with low techno-efficacy often lack confidence, exhibiting reduced satisfaction and negative emotions. Research confirms techno-efficacy effectively predicts behavioral performance in domain-specific tasks (Newman et al., 2018).

When AI-enabled job characteristics change, techno-efficacy significantly shapes workers’ technological attitudes and emotional responses. As Nisafani et al. (2020) demonstrate, techno-efficacy influences reactions to technical stressors: high-efficacy individuals proactively confront technological challenges to reduce anxiety, while low-efficacy workers focus on technology’s negative impacts (e.g., job displacement fears). This triggers psychological stress, work insecurity, and heightened hindrance stress perception. Consequently, High techno-efficacy workers perceive AI-enabled job characteristics primarily as challenge stress. Low techno-efficacy workers perceive them primarily as hindrance stress. Thus, we propose:

**H4a:**  *Techno-efficacy strengthens the positive relationship between AI-enabled job characteristics and perceived challenge stress.*

**H4b:**  *Techno-efficacy weakens the positive relationship between AI-enabled job characteristics and perceived hindrance stress.*

**Figure 1:**
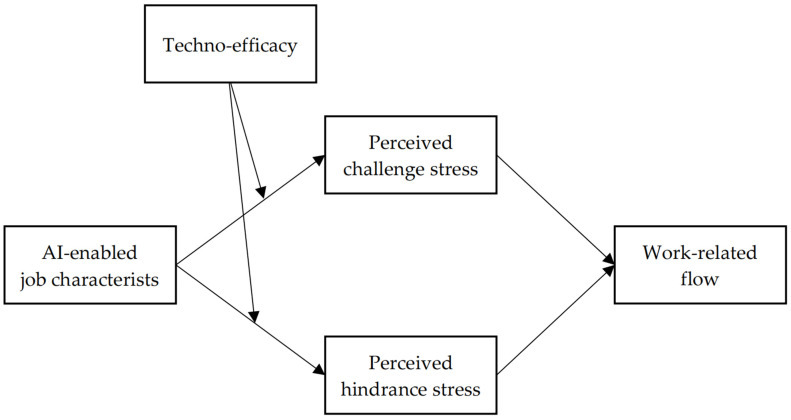
The theoretical conceptual model.

The theoretical model of this study is shown in Figure 1.

## 3. Material and Methods

### 3.1. Participants and Data Collection

This study focuses on frontline workers from Chinese manufacturing workplaces where AI technologies are implemented. The survey was conducted nationwide via the professional data collection platform “Credamo” (Company: Beijing Judshoots Technology Co., Ltd., Beijing, China). Data collection was conducted in [March–April, 2024], using the latest available version of the platform at that time. To ensure that the research subjects meet the sample characteristic requirements, this study includes a screening item: “Does your company apply AI technology to empower production (such as: using industrial robots, intelligent equipment, etc., to achieve automated production and intelligent decision-making)?” Research subjects who select “Yes” will be distributed the formal questionnaire for completion. After collection and statistics, a total of 497 formal questionnaires were collected, of which 405 were valid, resulting in an effective recovery rate of 81.49%. In terms of gender, males are the majority, accounting for 62%; in terms of age, respondents aged 30 years old or younger and those between 31–40 years old are the most common, accounting for 32.6% and 52.1%, respectively. Collectively representing approximately 84.7% of respondents, this demographic—broadly categorized as comprising Millennial and Generation Z cohorts—constitutes the majority of the sample, a distribution that is representative of the current profile of frontline manufacturing workers in China. In terms of education, those with junior college and above are the majority, accounting for 89.4%; in terms of work experience, those with more than 5 years of experience account for the highest proportion, at 52.8%, followed by 3–5 years, accounting for 25.9%.

### 3.2. Measurements

The scales used in this study are all derived from mature scales widely used in existing research and are compiled based on the research content. The English scales follow the standard process of translation and back-translation and are appropriately modified in line with the local context (Brislin, 1986). Considering the characteristics of Chinese ideology and culture, the questionnaire uses a 6-point Likert scale to avoid the possible “moderate tendency” of Chinese respondents and encourage more explicit statements, where 1, 2, 3, 4, 5, and 6 represent “Strongly Disagree,” “Disagree,” “Slightly Disagree,” “Slightly Agree,” “Moderately Agree,” and “Strongly Agree”, respectively.

#### 3.2.1. AI-Enabled Job Characteristics

The measurement of this variable uses the scale developed by Verma and Singh (2022), which comprises five dimensions and a total of 13 items (as shown in Table 1): AI enabled Job Autonomy (AI-JA), AI enabled Skill Variety (AI-SV), AI enabled Job Complexity (AI-JC), AI enabled Specialization (AI-Sp), AI enabled Information Processing (AI-IP). In this study, the internal consistency coefficient (*α* coefficient) of the AI-enabled job characteristics scale is 0.86. Regarding scoring methods, existing research has shown that adding the scores of each dimension of the AI-enabled job characteristics model is superior to the calculation method of the AI-enabled job characteristics motivational potential score proposed by Hackman and Oldham (Fried & Ferris, 1987). Many empirical studies also consider the sum of the scores of each sub-dimension as the overall score of AI-enabled job characteristics (Wei et al., 2017). Therefore, this study also adopts this scoring method.

#### 3.2.2. Perceived Challenge–Hindrance Stress

The measurement of this variable uses the scale developed by Rodell and Judge (2009), which consists of 8 items (as shown in Table 2). Perceived challenge stress was measured using four items capturing workload, time urgency, job responsibility, and job complexity, demonstrating good internal consistency (*α* = 0.83). Perceived hindrance stress was assessed with four items covering red tape, role ambiguity, role conflict, and hassles, which also showed acceptable reliability (*α* = 0.78).

#### 3.2.3. Work-Related Flow

The measurement of this variable uses the scale developed by Bakker (2008), which consists of three dimensions: absorption, work enjoyment, and intrinsic work motivation, with a total of 13 items (as shown in Table 3). In this study, the internal consistency coefficient (*α* coefficient) of the work-related flow scale is 0.91.

#### 3.2.4. Techno-Efficacy

The measurement of this variable uses the scale developed by Rayburn et al. (2021), which consists of 4 items (as shown in Table 4). In this study, the internal consistency coefficient (*α* coefficient) of the techno-efficacy scale is 0.85.

#### 3.2.5. Control Variables

Referring to previous studies, this study includes gender, age, educational level, and working years as control variables (Lyu et al., 2021).

## 4. Results

### 4.1. Common Method Bias Test and Confirmatory Factor Analysis

This study collected self-reported data at a single time point. To enhance rigor and control for common method bias, we first conducted Harman’s single-factor test. All questionnaire items underwent unrotated factor analysis, yielding a variance explained of 25.84%, which is below the critical threshold of 50% (Podsakoff et al., 2003). This indicates common method bias is not severe in this study.

We assessed model-data fit using AMOS 24.0 for confirmatory factor analysis, as shown in Table 5. The five-factor model demonstrated optimal fit indices (*χ*^2^ = 1059.102, *df* = 655, *χ*^2^/*df* = 1.617, RMSEA = 0.039, CFI = 0.932, TLI = 0.927, SRMR = 0.045). Alternative factor models showed significantly worse fit, confirming strong discriminant validity among the five study variables.

### 4.2. Descriptive Statistics and Correlation Analysis

The means (M), standard deviations (SD), and correlation coefficients between variables are shown in Table 6. AI-enabled job characteristics positively correlated with perceived challenge stress (*r* = 0.427, *p* < 0.01) and perceived hindrance stress (*r* = 0.118, *p* < 0.05), perceived challenge stress is significantly positively correlated with work-related flow (*r* = 0.328, *p* < 0.01), and perceived hindrance stress is significantly negatively correlated with work-related flow (*r* = −0.200, *p* < 0.01). These align with theoretical expectations and support further analysis.

### 4.3. Hypothesis Testing

#### 4.3.1. Main Effect and Mediating Effect Tests

This study used SPSS27.0 software to conduct hierarchical regression analysis to test the proposed hypotheses, as shown in Table 7. After controlling for demographic variables (models 1, 3, 5), Model 4: AI-enabled job characteristics positively predicted perceived challenge stress (*β* = 0.418, *p* < 0.001); Model 6: AI-enabled job characteristics positively predicted perceived hindrance stress (*β* = 0.139, *p* < 0.05). Thus, hypotheses 1a and 1b were supported. Model 7: perceived challenge stress positively predicted work-related flow (*β* = 0.268, *p* < 0.001), perceived hindrance stress negatively predicted work-related flow (*β* = −0.279, *p* < 0.001). Thus, H2a and H2b were supported.

This study used the Bootstrap method with 5000 resamples to confirmed mediation, as shown in Table 8. The mediating effect of perceived challenge stress is significant (indirect effect = 0.18, 95%CI = [0.11, 0.26]); the mediating effect of perceived hindrance stress is also significant (indirect effect = −0.05, 95%CI = [−0.10, −0.01]), Both CIs excluded zero. These results indicate dual mediating pathways with opposing effects, and verifying H3a and H3b.

#### 4.3.2. Moderating Effect Tests

**Figure 2:**
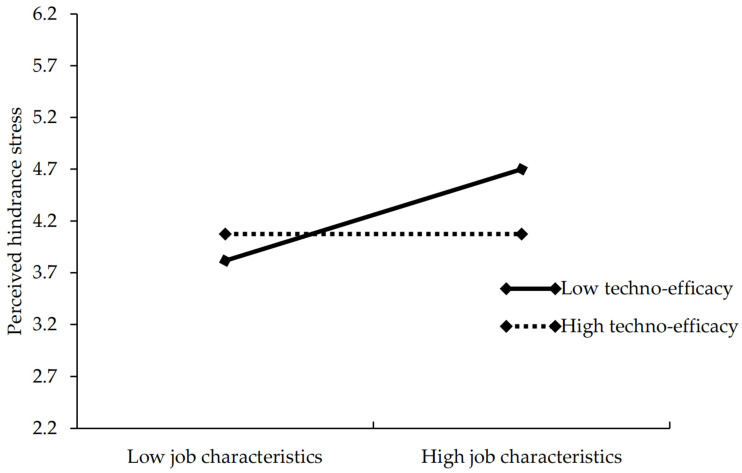
The moderating effect of techno-efficacy.

The moderating effect of techno-efficacy was further examined using hierarchical regression analysis, as shown in Table 9. Model 11 demonstrated that the interaction term between AI-enabled job characteristics and techno-efficacy has no significant effect on perceived challenge stress (*β* = −0.043, *p* > 0.05), thus, Hypothesis 4a is not supported. thus, Hypothesis 4a was not supported. In contrast, Model 14 indicated a significant negative effect of the interaction term on perceived hindrance stress (*β* = −0.147, *p* < 0.01), thus, hypothesis 4b is supported. To clarify the significance of the moderating effect, this study drew a moderation effect plot, as shown in Figure 2. The results revealed that when techno-efficacy is low, AI-enabled job characteristics significantly and positively predict perceived hindrance stress. However, when techno-efficacy is high, this positive relationship is attenuated. These findings suggest that lower levels of techno-efficacy strengthen the positive association between AI-enabled job characteristics and perceived hindrance stress.

### 4.4. Fuzzy-Set Qualitative Comparative Analysis

Hierarchical regression analysis, as a typical quantitative statistical analysis method based on linear correlation relationships, has limited ability to explain how multiple factors combine and what role each factor plays (Ma & Wang, 2024). In contrast, fuzzy-set qualitative comparative analysis (fsQCA) infer causality through set relations rather than linear correlation. This approach is better suited to identifying multiple concurrent conditions and the various causal paths that lead to work-related flow (Rasoolimanesh et al., 2021), Therefore, this study employs both hierarchical regression and fsQCA. The regression analysis established the overall correlation patterns among key variables and confirmed the presence of mediation and moderation effects. The fsQCA, on the other hand, uncovered heterogeneity in these relationships across different worker subgroups and identified multiple equifinal and non-linear conditional configurations leading to high work-related flow. Together, these methods provide mutual validation and complementary insights, offering a more comprehensive analytical perspective.

#### 4.4.1. Variable Selection and Calibration

This study selects five antecedent conditions, including AI-enabled job characteristics, perceived challenge stress, perceived hindrance stress, techno-efficacy, and working years. The selection of these antecedent conditions was primarily based on the following considerations. First, as confirmed by the preceding empirical tests, AI-enabled job characteristics, perceived challenge stress, perceived hindrance stress, and techno-efficacy have been theoretically and empirically supported in the existing literature regarding their influence on work-related flow. Second, in accordance with the approach adopted by L. Lu et al. (2024a), when conducting fsQCA as a supplementary analysis, control variables that are significantly correlated with the dependent variable are generally incorporated into the set of antecedent conditions for qualitative analysis. Additionally, as shown in Table 5, working years is significantly positively correlated with work-related flow (r = 0.196, *p* < 0.01), whereas other demographic variables showed no significant effect. Hence, working years was included as an antecedent condition.

Calibration was performed for the fsQCA. The continuous variables—AI-enabled job characteristics, perceived challenge stress, perceived hindrance stress, and techno-efficacy—were set to the mean, and calibration is carried out based on the three standards of complete membership at 5%, complete non-membership at 95%, and crossover at 50% (Ragin, 2008). Working years was calibrated as follows: less than 1 year = 0, 1–3 years = 0.33, 3–5 years = 0.67, and more than 5 years = 1. Necessity and sufficiency analyses were conducted for the antecedent variables of work-related flow, as shown in Table 10. Since no condition exceeded a necessity score of 0.9, none qualified as a necessary condition. Accordingly, a configurational analysis was performed to examine combinations of antecedent conditions.

#### 4.4.2. fsQCA Results

This study conducted analysis using fsQCA 3.0 software, with the consistency threshold set at 0.8 (Ragin, 2006), a minimum case threshold of 3, and PRI consistency at 0.75. The analysis yielded four antecedent configurations, as shown in Table 11. Among them, the overall consistency coefficient of the configurations was 0.81, with a coverage of 0.81, indicating that the four configurations cover 81% of the work-related flow sample.

*Configuration 1*: The antecedent configuration of S_1_ (~perceived challenge stress•~ perceived hindrance stress• techno-efficacy) indicates that low perceived hindrance stress combined with high techno-efficacy serves as the core condition for achieving work-related flow. In the face of work stressors, individual differences in stress perception influence coping strategy selection. Lower levels of perceived hindrance stress reduce workers’ focus on potential difficulties and obstacles. Simultaneously, techno-efficacy functions as a critical individual factor affecting stress perception—higher techno-efficacy corresponds to greater reduction in perceived hindrance stress, thereby facilitating work-related flow. This pattern partially aligns with the proposed mechanisms in H2b and H4b.

*Configuration 2*: The triggering type of configuration S_2_ includes two sub-patterns (S_2a_ and S_2b_), with the core condition being high perceived challenge stress and low perceived hindrance stress. In S_2a_ (working years• perceived challenge stress~ perceived hindrance stress), working years serves as an auxiliary condition that provides workers with unique advantages including work experience, capability, and social connections. Therefore, it is necessary to consider other factors that affect work-related flow. Working years, as a condition resource, may give workers unique advantages such as work experience, work ability, and social connections (Hobfoll et al., 2018), which enable workers to interpret AI-enabled job characteristic stress as challenge stress rather than hindrance, ultimately promoting work-related flow. In S_2b_ (AI-enabled job characteristics• perceived challenge stress•~ perceived hindrance stress), the auxiliary condition is AI-enabled job characteristics. When confronting AI-enabled changes in job characteristics, workers may experience both challenge and hindrance stress simultaneously. Only when AI-enabled job characteristics are predominantly perceived as challenging do they contribute to work-related flow, providing partial verification for hypotheses H3a and H3b.

*Configuration 3*: The antecedent configuration of S_3_ (working years• AI-enabled job characteristics• techno-efficacy) demonstrates that high values across all three conditions—working years, AI-enabled job characteristics, and techno-efficacy—form the core conditions for triggering work-related flow. As two types of work resources, extensive working years and favorable AI-enabled job characteristics equip workers with advantages that help overcome workplace difficulties while maintaining positive and confident work attitudes. At the same time, techno-efficacy, as an individual resource, complements these by stimulating intrinsic work motivation that leads to work immersion.

*Configuration 4*: The antecedent configuration of S_4_ (perceived challenge stress• perceived hindrance stress• techno-efficacy) reveals that high perceived challenge stress combined with high techno-efficacy constitutes the core conditions for achieving work-related flow, with high perceived hindrance stress acting as an auxiliary condition. This pathway confirms the driving role of stress perception and the moderating function of techno-efficacy in stress appraisal processes.

## 5. Discussion

### 5.1. Research Findings

Based on the cognitive appraisal theory of stress, this study examined the impact mechanism of AI-enabled job characteristics on work-related flow among 405 manufacturing workers. Using hierarchical regression and fuzzy-set qualitative comparative analysis (fsQCA), the findings reveal that: (1) Perceived challenge stress and perceived hindrance stress mediate the relationship between AI-enabled job characteristics and work-related flow. Perceived challenge stress shows a significant positive correlation with work-related flow, whereas perceived hindrance stress is significantly negatively correlated with it. It should be noted that this mediating pathway reflects a conditional relationship among variables rather than a deterministic causal sequence; (2) Techno-efficacy negatively moderates the relationship between AI-enabled job characteristics and perceived hindrance stress, but does not significantly moderate the link between AI-enabled job characteristics and perceived challenge stress; (3) The fsQCA identified four distinct antecedent configurations that lead to a high level of work-related flow. Among these, configuration S_3_ demonstrates greater coverage than the others, suggesting stronger explanatory power. This indicates that the combination of working years, AI-enabled job characteristics, and techno-efficacy accounts for more instances of work-related flow. Additionally, working years is confirmed as a key factor influencing work-related flow.

The study did not support H4a. Potential reasons may include the following: according to cognitive appraisal theory of stress, an individual’s stress perception is influenced not only by personal factors but also by situational elements. In the context of AI applications, workers may place greater emphasis on external technological environments—such as leadership style and organizational climate—which convey support and encouragement (Jiang et al., 2025). These situational factors are more likely to enhance an individual’s sense of competence and perception of challenge when confronted with stressors. Similarly to the findings of Du and Chen (2023), we posit that techno-efficacy functions more effectively as a resource for mitigating loss (i.e., buffering against hindrance stress) than for actively promoting gain. Such resources tend to be experiential, ambiguous, and lack contextual specificity, making them relatively abstract (Sun & Chen, 2024). While they help individuals maintain confidence in mitigating the adverse effects of AI technology and reduce perceived hindrance stress (Bandura, 2012), they are less effective than gain-oriented resources (e.g., leadership support, clear task feedback, or career development opportunities) in facilitating active pursuit of goals and overcoming challenges (Bakker & Demerouti, 2017; Van Den Broeck et al., 2016). This aligns with the theoretical notion that resources serve distinct motivational functions, with some being more pivotal for loss-prevention and others for active gain-promotion (Hobfoll et al., 2018). Therefore, the role of techno-efficacy in promoting perceived challenge stress remains limited.

### 5.2. Theoretical Contributions

This study makes several theoretical contributions. First, by introducing AI into the research on worker psychology and behavior, it expands the contextual understanding of how AI-enabled job characteristics influence work-related flow among manufacturing workers. Previous studies on AI-enabled job characteristics have largely been dominated by the Job Demands-Resources (JD-R) model and the AI-enabled job characteristics Model (JCM), both developed within Western industrial contexts. Their applicability to China—given its distinct sociocultural and economic conditions—requires further validation. Moreover, while some scholars argue that a realistic AI-enabled job characteristics model should adapt to different professions and work environments (Lin, 2021), empirical research in this vein remains limited. Against this backdrop, this study examines five AI-enabled job characteristics—AI-enabled job autonomy, AI-enabled skill variety, AI-enabled job complexity, AI-enabled specialization, AI-enabled information processing—and their effects on work-related flow, thereby broadening the contextual scope of AI-enabled job characteristics research, particularly within the Chinese manufacturing sector (Wang et al., 2023) and among the rising generations of workers who are more adaptable to digital transformation but also face greater job mobility and skill obsolescence (Lyons et al., 2015).

Second, drawing on the cognitive appraisal theory of stress, this research elucidates the dual-path mechanism through which AI-enabled job characteristics exert both positive and negative effects on work-related flow in AI-enhanced work settings. This offers a more holistic perspective on individual-level AI impacts. Previous studies indicate that AI adoption yields mixed outcomes: workers may experience job displacement, heightened skill demands, increased tension, anxiety, burnout, and insecurity (Srivastava et al., 2015), while also benefiting from reduced repetitive tasks and enhanced innovation and achievement (H. Zhang et al., 2023). Building on this evidence, our study underscores the role of stress appraisal and acknowledges the dual nature of job characteristic stress.

Furthermore, the results demonstrate that higher techno-efficacy serves as a key boundary condition that reduces perceived hindrance stress. This aligns with calls for closer examination of context-specific self-efficacy (Rayburn et al., 2021). Techno-efficacy functions as a work resource that facilitates task performance, promotes proactive skill acquisition, and strengthens work engagement (Delpechitre et al., 2019). This study reveals that such benefits occur specifically through the mitigation of hindrance stress. As an internal resource, techno-efficacy helps buffer the adverse effects of job characteristic stress, supporting the attainment of work-related flow for manufacturing workers.

Finally, through the application of a configurational approach, this study identifies multiple pathways leading to workers’ work-related flow. It responds to scholarly recommendations for methodological pluralism by combining hierarchical regression with fsQCA to examine antecedent conditions and outcome patterns (Wellman et al., 2023). Notably, working years—initially a control variable significantly correlated with work-related flow—was incorporated as an antecedent, revealing meaningful causal configurations. These findings enrich AI-enabled job characteristics research by integrating both linear and set-theoretic analytical perspectives.

### 5.3. Practical Implications

The findings of this study offer important implications for management practice. In the context of the intelligent transformation of global manufacturing, the deep application of AI technology is reshaping production models and work environments. With the widespread adoption of tools such as smart robots, automated production lines, and big data analytics, the nature of manufacturing workers’ tasks has shifted from traditional repetitive operations to more complex responsibilities requiring higher technical proficiency and decision-making capabilities. As a result, job characteristic stress has emerged as a key factor influencing work motivation and performance. Accordingly, this paper proposes the following management recommendations:

Implement differentiated stress perception management. Stressors possess dual attributes of being both challenging and hindering, and workers’ cognitive appraisal of stress directly influences their behavioral outcomes. On one hand, organizations should prioritize job design by proactively incorporating challenging elements (e.g., dynamically matching task requirements with workers’ capabilities through a “challenge-skill balance” model) to stimulate intrinsic motivation and foster a sense of achievement, thereby promoting deep work immersion. On the other hand, systematic assessment and support mechanisms should be established to mitigate hindering stress. For instance, multi-source evaluation methods (e.g., 360-degree feedback) can be introduced to comprehensively identify obstacles in tasks; an intelligent issue-response platform can be built to enable real-time diagnosis and support for technical difficulties; and a mental health support system can be set up to reduce uncertainty and anxiety induced by AI applications through regular counseling and team-building activities.

Strengthen the cultivation of workers’ technical self-efficacy. This study confirms that technical self-efficacy is a key mechanism for reducing the perception of hindering stress. To enhance workers’ confidence in meeting AI-related demands, organizations should establish systematic and ongoing training systems, including setting up skill development centers, organizing hands-on training sessions, and technical competitions to ensure continuous alignment between workers’ capabilities and job requirements. For workers with low technical self-efficacy, a “technical mentorship program” can be implemented, where highly skilled workers provide technical guidance and emotional support. This helps them gradually accumulate successful experiences, build technical confidence, and thereby alleviate stress perceptions arising from AI integration.

Leverage the buffering and enabling role of organizational support and leadership. Research indicates that relying solely on workers’ personal resources is insufficient to effectively transform challenging stress; organizations must provide complementary support resources (Osei et al., 2024; Wallace et al., 2009). Enterprises can establish AI technical support centers offering 24 h online consultations and remote assistance, while systematically integrating learning resources (e.g., case libraries, operational guidelines) to lower the barriers to technology adoption. Simultaneously, leaders should play a pivotal guiding role: through regular communication, personalized growth plans, and targeted resource support, they can help workers navigate the challenges posed by AI technologies. Positive leadership styles (e.g., charismatic or entrepreneurial leadership) can also significantly enhance workers’ positive appraisal of challenging stress, thereby improving their adaptation and performance under technological pressure (M. A. LePine et al., 2016; Jiang et al., 2025).

## 6. Limitations and Future Research

This study has several limitations that should be acknowledged. First, the use of the online survey platform for participant recruitment may introduce self-selection bias. For instance, workers who are more technologically adept or comfortable in digital environments may be more inclined to take part, which could reduce the representativeness of the sample—especially for manufacturing workers with lower levels of technological adaptation. Future studies should consider more direct sampling approaches, such as organizational partnerships or onsite surveys, to improve generalizability. Second, all data were collected through self-report questionnaires, which are susceptible to common method variance. In addition, the cross-sectional design prevents causal inference. Future research could adopt longitudinal designs with multiple time points and incorporate data from multiple sources to enhance measurement accuracy. Finally, this study examined only the moderating role of an individual trait—techno-efficacy—in the relationship between AI-enabled job characteristics and outcomes. Previous research suggests that situational factors, such as organizational support and leadership style, also significantly influence workers’ stress perceptions (Yi et al., 2023). Thus, future studies should develop a more comprehensive theoretical model that incorporates both personal and contextual factors to better understand how AI-enabled job characteristics affect work-related flow.

## 7. Conclusions

As artificial intelligence becomes deeply embedded in the fabric of modern manufacturing, understanding its psychological impact on the workforce is critical for successful organizational integration. This study sought to move beyond a simplistic view of AI’s effects by investigating the dual-path mechanism through which AI-enabled job characteristics influence work-related flow. Grounded in the challenge–hindrance stress framework, our research provides a more nuanced perspective on worker adaptation to AI-driven workplaces.

Through a multi-method approach combining empirical analysis and fsQCA, our findings confirm that AI-enabled job characteristics are a double-edged sword. They serve as a significant source of both challenge stress, which enhances work-flow by promoting engagement and mastery, and hindrance stress, which diminishes it by inducing strain and uncertainty. A pivotal insight from our study is the role of techno-efficacy as a critical boundary condition that buffers workers against the detrimental effects of hindrance stress, thereby helping to unlock the positive potential of AI.

These findings offer a valuable lens for organizations and managers to view the human side of AI integration. Rather than treating AI as a purely technological upgrade, managers must recognize its profound psychological impact on workers. By implementing differentiated strategies that amplify challenge appraisals—through thoughtful job redesign and leadership support—while simultaneously mitigating hindrances—via targeted training and robust technical support—organizations can foster a more adaptive and resilient workforce.

Ultimately, this research underscores that the path to leveraging AI for productivity gains is inextricably linked to its impact on worker experience. By cultivating an environment that reduces threat and promotes growth, organizations can not only enhance work-flow and well-being but also achieve the synergistic goal of harmonizing technological advancement with human flourishing in the era of intelligent manufacturing.

## Figures and Tables

**Table 1:** Reliability analysis of AI-enabled job characteristics.

Dimensions	Items
AI-JA	AIS helps to make decisions in real-time.
	AIS helps to make decisions about what methods I should use to complete my work.
	AIS helps to perform a variety of tasks in a short time.
	AIS helps to get direct and clear information about the effectiveness (i.e., quality and quantity) of my job performance.
AI-SV	Jobs using AIS require tracking of more than one thing at a time.
	Jobs using AIS require a variety of skills to complete the work.
AI-JC	Using AIS, I can do multiple tasks/activities at a time.
	Using AIS, my job becomes comparatively simple.
AI-Sp	Jobs using AIS require high specialization of purpose, tasks, or activities.
	Jobs using AIS require highly specialized knowledge and skills.
	Jobs using AIS require in-depth advanced technological expertise.
AI-IP	Jobs using AIS require a lot of information analysis.
	Jobs using AIS require to engage in less thinking.

Abbreviations: AIS, Artificial Intelligence system.

**Table 2:** Reliability analysis of perceived challenge–hindrance stress.

Dimensions	Items
Perceived Challenge Stress	Today, my job has required me to work very hard
	Today, I have experienced severe time pressures in my work
	Today, I’ve felt the weight of the amount of responsibility I have at work
	Today, my job has required me to use a number of complex or high-level skills
Perceived Hindrance Stress	Today, I have had to go through a lot of red tape to get my job done
	Today, I have not fully understood what is expected of me
	Today, I have received conflicting requests from two or more people
	Today, I have had many hassles to go through to get projects/assignments done

**Table 3:** Reliability analysis of work-related flow.

Dimensions	Items
Absorption	When I am working, I think about nothing else
	I get carried away by my work
	When I am working, I forget everything else around me
	I am totally immersed in my work
Work Enjoyment	My work gives me a good feeling
	I do my work with a lot of enjoyment
	I feel happy during my work
	I feel cheerful when I am working
Intrinsic Work Motivation	I would still do this work, even if I received less pay
	I find that I also want to work in my free time
	I work because I enjoy it
	When I am working on something, I am doing it for myself
	I get my motivation from the work itself, and not from the reward for it

**Table 4:** Reliability analysis of Techno-efficacy.

Dimensions	Items
Techno-efficacy	I have the resources necessary to use AI
	I have the knowledge necessary to use AI
	AI is compatible with other systems I use
	I receive assistance with AI difficulties

**Table 5:** Results of confirmatory factor analysis.

Model	*χ* ^2^	*df*	*χ*^2^/*df*	RMSEA	CFI	TLI	SRMR
Five factors: *J*, *C*, *H*, *T*, *W*	1059.102	655	1.617	0.039	0.932	0.927	0.045
Four factors: *J + W*, *C*, *H*, *T*	2064.447	659	3.133	0.073	0.763	0.747	0.097
Three factors: *J + W + T*, *C*, *H*	2375.699	662	3.589	0.08	0.711	0.693	0.097
Two factors: *J + H + W*, *C + T*	2987.032	664	4.499	0.093	0.608	0.585	0.115
Single factor: *J + C + H + T + W*	3265.350	665	4.910	0.098	0.561	0.536	0.113

Note: *N* = 405; *χ*^2^/*df*, chi-square/degrees of freedom. Abbreviations: *J*, AI-enabled job characteristics; *C*, perceived challenge stress, *H*, perceived hindrance stress; *W*, work-related flow; *T*, techno-efficacy; RMSEA, root mean square error of approximation; CFI, Comparative fit index; TLI, Tucker–Lewis index; SRMR, Standardized root mean square residual.

**Table 6:** Means, SDs, and correlation analysis.

Variable	*M* (*SD*)	1	2	3	4	5	6	7	8	9
1. Gender	1.38 (0.486)	1								
2. Age	1.83 (0.671)	−0.071	1							
3. Educational level	3.17 (0.888)	0.035	−0.300 **	1						
4. Working years	3.29 (0.866)	−0.083	0.482 **	−0.04	1					
5. AI-enabled job characteristics	4.71 (0.520)	0.061	0.072	0.022	0.145 **	1				
6. Perceived challenge stress	4.29 (0.775)	0.019	−0.009	0.081	0.114 *	0.427 **	1			
7. Perceived hindrance stress	3.64 (0.730)	−0.037	−0.029	−0.048	−0.087	0.118 *	0.220 **	1		
8. Work-related flow	4.40 (0.782)	−0.024	0.093	0.088	0.196 **	0.349 **	0.328 **	−0.200 **	1	
9. Techno-efficacy	4.55 (0.862)	0.012	0	0.255 **	0.209 **	0.501 **	0.317 **	−0.020	0.527 **	1

Note: ** *p* < 0.01; * *p* < 0.05. Abbreviations: *M*, means; *SD*, standard deviations.

**Table 7:** Main effect and intermediate effect test.

Variables	Work-Related Flow			Perceived Challenge Stress		Perceived Hindrance Stress	
	Model 1	Model 2	Model 7	Model 3	Model 4	Model 5	Model 6
Control variable							
Gender	−0.010	−0.034	−0.047	0.025	−0.006	−0.042	−0.053
Age	0.037	0.032	0.047	−0.058	−0.065	−0.004	−0.006
Educational level	0.107 *	0.097 *	0.066	0.069	0.056	−0.051	−0.055
Working years	0.181 **	0.134 *	0.080	0.147 **	0.086	−0.091	−0.111
Independent variable							
AI-enabled job characteristics		0.328 ***	0.254 ***		0.418 ***		0.139 *
Intermediate variable							
Perceived challenge stress			0.268 ***				
Perceived hindrance stress			−0.279 ***				
*R* ^2^	0.049	0.153	0.260	0.023	0.193	0.012	0.031
*F*	5.109 **	14.424 ***	19.927 ***	2.390 *	19.124 ***	1.211	2.533 *
Δ*R*^2^	0.049	0.104	0.073	0.023	0.170	0.012	0.019
Δ*F*	5.109 **	49.224 ***	38.950 ***	2.390 *	84.076 ***	1.211	7.743 *

Note: *** *p* < 0.001; ** *p* < 0.01; * *p* < 0.05.

**Table 8:** Mediating effect test.

	Effect	Boot SE	Boot LLCI	Boot ULCI
Direct effect	0.399	0.072	0.256	0.540
Indirect effects of perceived challenge stress	0.180	0.040	0.105	0.262
Indirect effects of perceived hindrance stress	−0.052	0.023	−0.099	−0.008
Total stress	0.525	0.070	0.388	0.663

Abbreviations: Boot SE, Bootstrap standard error; Boot LLCI, Bootstrap lower level of confidence interval; Boot ULCI, Bootstrap upper level of confidence interval.

**Table 9:** Moderating effect of techno-efficacy.

Variables		Perceived Challenge Stress			Perceived Hindrance Stress		
		Model 9	Model 10	Model 11	Model 12	Model 13	Model 14
Control variable	Gender	0.025	−0.004	−0.002	−0.042	−0.054	−0.047
	Age	−0.058	−0.059	−0.058	−0.004	−0.009	−0.005
	Educational Level	0.069	0.029	0.030	−0.051	−0.036	−0.035
	Working years	0.147 **	0.067	0.067	−0.091	−0.097	−0.099
Independent variable	AI-enabled job characteristics		0.364 ***	0.360 ***		0.176 **	0.161 **
Moderating variable	Techno-efficacy		0.113 *	0.103		−0.079	−0.114
Interaction term	AI-enabled job characteristics × Techno-efficacy			−0.043			−0.147 **
*R* ^2^		0.023	0.202	0.204	0.012	0.035	0.055
*F*		2.390 *	16.777 ***	14.494 ***	1.211	2.397 *	3.270 **
Δ*R*^2^		0.023	0.179	0.002	0.012	0.023	0.020
Δ*F*		2.390 *	44.513 ***	0.853	1.211	4.725 **	8.248 **

Note: *** *p* < 0.001; ** *p* < 0.01; * *p* < 0.05.

**Table 10:** Analysis results of necessary conditions for work-related flow.

Variable Name	High Work-Related Flow		Low Work-Related Flow	
	Consistency	Coverage	Consistency	Coverage
Working years	0.887	0.600	0.824	0.524
~Working years	0.295	0.640	0.370	0.755
AI-enabled job characteristics	0.744	0.748	0.590	0.559
~AI-enabled job characteristics	0.561	0.592	0.734	0.730
Perceived challenge stress	0.707	0.763	0.564	0.574
~Perceived challenge stress	0.605	0.595	0.768	0.712
Perceived hindrance stress	0.633	0.615	0.729	0.668
~Perceived hindrance stress	0.658	0.720	0.580	0.599
Techno-efficacy	0.811	0.770	0.579	0.517
~Techno-efficacy	0.492	0.553	0.742	0.787

Notes: “~” means logical operator NOT.

**Table 11:** Antecedent condition configuration of work-related flow.

Configuration	Work-Related Flow				
	*S*_1_	*S*_2*a*_	*S*_2*b*_	*S*_3_	*S*_4_
Working years		●		⚫	
AI-enabled job characteristics			●	⚫	
Perceived challenge stress	⊗	⚫	⚫		⚫
Perceived hindrance stress	⊗	⊗	⊗		●
Techno-efficacy	⚫			⚫	⚫
Original coverage	0.403	0.437	0.400	0.620	0.454
Unique coverage	0.046	0.032	0.013	0.056	0.042
Unique consistency	0.876	0.864	0.889	0.847	0.855
Overall coverage	0.811				
Overall consistency	0.808				

Note: ⚫ means the presence of core conditions; ● means the presence of peripheral conditions; ⊗ means that the absence of core condition.

## Data Availability

The data are not publicly available due to privacy or ethical restrictions. The data presented in this study are available on request from the corresponding author.
